# Effects of Aqueous Extract of *Berberis integerrima* Root on Some Physiological Parameters in Streptozotocin-Induced Diabetic Rats 

**Published:** 2013

**Authors:** Hossein Ashraf, Reza Heidari, Vahid Nejati, Minoo Ilkhanipoor

**Affiliations:** *Department of Biology, Faculty of Sciences, Urmia University, Urmia, Iran. *

**Keywords:** Berberis Integerrima, Streptozotocin, Hypoglycemic, Hypolipidemic, Antioxidant

## Abstract

Diabetes mellitus is a common endocrine disorder. Anti-diabetic agents from natural and synthetic sources are available for the treatment of this disease. Berberis integerrima is a medicinal shrub used in conventional therapy for a number of diseases. The aim of the present study was to investigate the effects of aqueous extract of Berberis integerrima root (AEBI) on some physiological parameters in normal and streptozotocin-induced (STZ-induced) diabetic male Wistar rats. STZ-induced diabetic rats showed significant increases in the levels of blood glucose, triglycerides (TG), total cholesterol (TC), low density lipoprotein LDL-cholesterol (LDL-C), creatinine (Cr), urea, alanine aminotransferase (ALT), aspartate aminotransferase (AST), alkaline phosphatase (ALP), total bilirubin while body weight, high density lipoprotein HDL-cholesterol (HDL-C) and total protein levels were significantly decreased compared to normal rats. Treatment of diabetic rats with different doses of aqueous extract of Berberis integerrima root (250 and 500 mg/Kg bw) resulted in a significant decrease in blood glucose, triglycerides, cholesterol, LDL-cholesterol, ALT, AST, ALP, total bilirubin, creatinine and urea while HDL-cholesterol and total protein levels were markedly increased after six weeks compared to untreated diabetic rats. The effects of the AEBI at dose of 500 mg/Kg in all parameters except blood glucose (similar) is more than to the standard drug, glibenclamide (0.6 mg/Kg, p.o.). The results of this study indicate that the tested aqueous extract of Berberis integerrima root possesses hypoglycemic, hypolipidemic and antioxidant effects in STZ-induced diabetic rats.

## Introduction

Diabetes Mellitus is the heterogeneous metabolic disorder characterized by altered carbohydrate, lipid and protein metabolism (1). More than 170 million people worldwide have diabetes and this number is set to be increase up to 360 million by 2030 (2). In Iran, approximately 2 million adult people have been diagnosed with diabetes and about 4.4 million of them have impaired fasting glucose (3). Although the main and effective treatment for diabetes mellitus is currently using insulin and hypoglycemic factors, these compounds have undesirable effects such as increased fat deposits, loss of fat tissue at the injection site and causing a hypoglycemic shock and having no effects on the trends of debilitating complication of diabetes in the long-term. Given the increase in human knowledge about the heterogeneity of this illness, there is a need to find effective combinations with fewer side effects in the treatment of diabetes (4). In addition, there are several forms of dyslipidemia in patients with diabetic mellitus. Due to the heart-vascular risks induced by hyperglycemia and hyperlipidemia, lipid disorders should be quickly diagnosed and treated as a part of diabetes comprehensive treatment. The most common pattern of dyslipidemia is increased triglycerides and decreased HDL cholesterol (5). Although medical plants and their ingredients have been considered a long time in the treatment of diabetes mellitus, there are not reliable and valid studies on their absolute effectiveness (6). Berberis integerrima (Berberidaceae) is an important medicinal shrub, to a height of about 4 m, with thick and leathery leaves, a cluster inflorescence with 2-5 cm length and berry fruit with 7-8 mm length. This plant grows in most regions of Iran, especially in northern and northeast regions of the country. The harvest time is in November. Due to having secondary metabolites such as Berberine, Oxyacanthine, Berbamine, Palmatine, Jatrorrhizine, Columbamine and Berberubine, this plant has much medicinal properties (7) and is used in the treatment of gastrointestinal disease, bleeding, swollen gums teeth, sore throat, fever, bile, malaria, hepatitis, inflammation and diarrhea. In addition, it has an important role in reducing blood cholesterol (7-10). Berberine is demonstrated to reduce serum cholesterol, triglyceride and LDL-cin subjects with dyslipidemia in animals (52).

Since few studies are available on anti-diabetic properties of this plant (B. integerrima), we examined the effects of aqueous extracts of Berberis integerrima root on some physiological parameters in streptozotocin-induced diabetic rats and compared these effects with sulfonylurea drugs like glibenclamide in a period of 6 weeks.

## Experimental

Streptozotocin (STZ) was purchased from Sigma Chemical Co. (St. Louis, MO, USA). All the remaining chemicals were of highest commercially available grades.

*Plant material*

Wild samples of Barberry root (*Berberis integerrima*) were collected from suburb Bavanat City (Fars Province, Iran) during November and December 2011 and identified by the Botany Department of Urmia University. A voucher specimen of the plant was deposited in the herbarium of the Faculty of Sciences, Urmia University, Urmia, Iran (No. 9059).

*Preparation of aqueous extract*

Roots were dried in the shade after being washed with cold water and were then powdered by using dry grinder and passed through the sieve. The aqueous extract was prepared by cold maceration of 150 g of powdered root barks in 500 mL of distilled water for 72 h. Then, the extract was filtered, concentrated, dried *in-vacuo *(yield 10 g) and the residue was stored in a refrigerator at 2-8°C for use in subsequent experiments (11).

*Animals*

Male Wistar rats weighing approximately 180-220 g (obtained from the central animal house of the Tehran Pasteur Institute, Tehran, Iran) were housed in an air-conditioned room under a 12-h light-dark cycle. Animals were allowed free access to tap water and standard laboratory rat food. All experimental procedures involving animals were approved by the Animal Research Ethics Committee of Urmia University of Faculty of Sciences, Urmia, Iran.

*Acute toxicity study*

Acute toxicity study of *aqueous *extract of *Berberis integerrima root *was determined as per the OECD guideline No. 423 (Acute Toxic Class Method). It was observed that test extract was not lethal to the rats even at 2500 mg/Kg dose. Hence, 1/10^th^ (250 mg/Kg) and 1/5^th^ (500 mg/Kg) of this dose were selected for further study (12).

*Experimental induction of diabetes*

Diabetes was induced in rats by intraperitoneal (IP) injection of streptozotocin (STZ) at a dose of 65 mg/Kg bw, dissolved in 0.1 cold citrate buffer (pH = 4.5) (13). Blood samples were taken from the tail vein 72 h after the STZ injection to measure the blood glucose levels by ACCU-Check glucose meter. Just animals with fasting blood glucose levels (after fasting for 12 h) over 300 mg/dL were considered diabetic and used for the further study (14).

*Experimental design*

All animals were randomly divided into eight groups with six animals in each group.

1. Normal control treated with normal saline (10 mL/Kg).

2. Normal rats treated with aqueous extract of Berberis integerrima root (250 mg/Kg body weight).

3. Normal rats treated with aqueous extract of Berberis integerrima root (500 mg/Kg body weight).

4. Normal rats treated with Glibenclamide (0.6 mg/Kg body weight)

5. Diabetic control treated with normal saline (10 mL/Kg).

6. Diabetic rats treated with aqueous extract of Berberis integerrima root (250 mg/Kg body weight).

7. Diabetic rats treated with aqueous extract of Berberis integerrima root (500 mg/Kg body weight).

8. Diabetic rats treated with Glibenclamide (0.6 mg/Kg body weight).

Animals were treated daily by gavage for 6 weeks and the experimental period for each rat was 6 weeks.

*Blood collection*

At the end of the study, animals were fasted overnight and anesthetized with chloroform (Pharmaceutical Partners of Japan). Blood samples were collected from the animal’s hearts and the serum was separated by centrifugation (3000 rpm at 4°C for 15 min) and stored at - 30°C for different biochemical analysis.

*Estimation of body weight*

The body weight in experimental animals was determined before the study and at 2, 4 and 6 weeks after it by a digital balance. These weights were determined at the same time during the morning.

*Estimation of blood glucose*

Throughout the 6-week treatment period, fasting (12 h) blood glucose was measured before the study, and at 2, 4 and 6 weeks after it on lateral tail vein blood samples using an ACCU-Check glucose meter (Roche, Mannheim, Germany)

*Estimation of some serum physiological parameters*

All biochemical parameters in serum concentration including of triglycerides, cholesterol, high density lipoprotein HDL-cholesterol (HDL-C), low density lipoprotein LDL-cholesterol (LDL-C), total protein, creatinine, urea, alanine aminotransferase (ALT), aspartate aminotransferase (AST), alkaline phosphatase (ALP) and total bilirubin determined with the use of commercially available enzyme kits (Pars Azmoon, Tehran, Iran) and using an automatic analyzer (Architect c8000 Clinical Chemistry System, USA). LDL cholesterol (LDLC) was estimated by Frydvald method: LDL cholsterol = total cholesterol - HDL cholesterol - (Triglyceride / 5).

*Statistical analysis*

All the reported data are expressed as mean ± SEM. Statistical analysis was performed using one‐way ANOVA followed by Tukey’s multiple tests using 18^th^ version of the computer software. The values were considered statistically significant when p*-*value was less than 0.05 compared to the respective control.

## Results

*Blood glucose*

The hypoglycemic effect of AEBI and glibenclamide on the fasting blood sugar levels of normal and diabetic rats is shown in Table 1.

**Table 1:** Effect of glibenclamide and AEBI on the blood glucose in normal and diabetic rats

**Group (n = 6)**	**Treatment**	**Dose (mg/Kg)**	**Blood glucose level (mg/dL)**			
			**Week 0**	**Week 2**	**Week 4**	**Week 6**
1	N+C	10 mL/Kg	89.40 ± 5.98	89.20 ± 4.50	98.20 ± 4.52	93.80 ± 3.35
2	N+AEBI	250	89.00 ± 4.04	69.00 ± 4.04^b^	77.40 ± 3.24^a^	86.40 ± 3.31
3	N+AEBI	500	97.40 ± 2.48	61.60 ± 2.60^b^**	82.20 ± 3.03	94.00 ± 2.40
4	N+G	0.6	89.20 ± 5.57	67.20 ± 3.02^b^*	81.60 ± 4.44	90.80 ± 5.34
5	D+C	10 mL/Kg	89.40 ± 4.73	316.00 ± 2.16^#^**	327.00 ± 3.92^#^**	337.20 ± 7.53^#^**
6	D+AEBI	250	97.20 ± 3.13	220.60 ± 3.09^d^**	175.4 ± 5.50^d^**	162.40 ± 2.61^d^**
7	D+AEBI	500	89.80 ± 4.27	181.80 ± 2.26^d^**	145.20 ± 2.74^d^**	110.40 ± .92^d^
8	D+G	0.6	87.20 ± 6.41	186.40 ± 2.37^d^**	147.40 ± 3.60^d^**	113.00 ± 3.43^d^**

AEBI: Aqueous extract of Berberis integerrima; N: normal; C: control; G: glibenclamide; D: diabetic; Values are presented as mean ± SEM; n = 6 in each group. One way ANOVA followed by Tukey test. a p < 0.05 and bp < 0.01. Normal treated rats were compared with Normal control Rats. #p < 0.01 Diabetic control Rats were compared with Normal control Rats. cp < 0.05 and dp < 0.01 Diabetic treated rats were compared with Diabetic control rats on corresponding day;*p < 0.05 and**p < 0.01 compared to 0 value

 The results clearly indicated that the AEBI (250 and 500 mg/Kg bw) or glibenclamide (0.6 mg/Kg bw) shows a significant hypoglycemic activity in normoglycemic rats by 22.48, 36.76, and 24.67% respectively in 2 weeks of treatment, however, at the end of 6^th^ week, it was increased to achieve the normal blood sugar levels that are 86.40 ± 3.31 mg/dL, 94.00 ± 2.40 mg/dL and 90.80 ± 5.34 mg/dL respectively. The administration of STZ (65 mg/Kg, IP) led to about 3.5-fold elevation of fasting blood glucose levels. Diabetic rats treated with AEBI (250 and 500 mg/Kg bw) or glibenclamide (0.6 mg/Kg bw) showed a significant decrease in blood glucose level during the 6 weeks of treatment. The value of blood glucose levels of diabetic rats in the 6 weeks of treatment in comparison with diabetic control are 51.83, 67.25 and 66.48%, respectively.

*Changes in body weight*

As shown in Table 2, normal control animals were found to be stable in their body weight. Diabetic rats treated by 250 mg/Kg bw of AEBI showed a little reduction in body weight by percentage 3.84% during 6 weeks but diabetic rats treated by 500 mg/Kg bw of AEBI or glibenclamide (0.6 mg/Kg bw) showed a negligible increase in the body weight by 3.03 and 1.7% respectively. While normal rats treated by AEBI (250 and 500 mg/Kg bw) or glibenclamide (0.6 mg/Kg bw) showed a little reduction in the body weight by 16.02, 17.86 and 20.28% respectively, the body weight of diabetic control group was decreased to 33.3% during 6 weeks.

**Table 2:** Effect of glibenclamide and AEBI on body weight in normal and diabetic rats

**Group (n = 6)**	**Treatment**	**Dose (mg/Kg)**	**Average body weight (g)**			
			**Week 0**	**Week 2**	**Week 4**	**Week 6**
1	N+C	10 mL/Kg	193.20 ± 6.58	211.56 ± 4.74	233.34 ± 3.20**	247.62 ± 1.87**
2	N+AEBI	250	204.08 ± 6.03	216.28 ± 5.22	226.90 ± 4.78	236.78 ± 3.58**
3	N+AEBI	500	200.94 ± 3.47	210.20 ± 2.91	219.76 ± 3.34	236.86 ± 3.18**
4	N+G	0.6	197.86 ± 6.20	212.32 ± 4.41	223.64 ± 4.02*	238.80 ± 2.41**
5	D+C	10 mL/Kg	206.84 ± 3.35	155.28 ± 4.97^#^**	144.90 ± 2.01^#^**	137.98 ± 2.24^#^**
6	D+AEBI	250	195.04 ± 4.67	183.44 ± 2.88^d^	180.96 ± 3.74^d^	187.56 ± 3.73^d^
7	D+AEBI	500	194.36 ± 1.78	181.90 ± 1.62^d^	183.52 ± 5.97^d^	200.22 ± 2.78^d^
8	D+G	0.6	199.94 ± 1.72	184.30 ± 4.08^d^	193.46 ± 5.53^d^	203.52 ± 5.61^d^

AEBI: Aqueous extract of Berberis integerrima; N: normal; C: control; G: glibenclamide; D: diabetic; Values are presented as mean ± SEM. n = 6 in each group. One way ANOVA followed by Tukey test. a p < 0.05 and bp < 0.01 Normal treated rats were compared with Normal control rats. #p < 0.01 Diabetic control rats were compared with Normal control Rats. cp < 0.05 and dp < 0.01 Diabetic treated rats were compared with Diabetic control Rats on corresponding day;*p < 0.05 and**p < 0.01 compared to 0 value

*Serum lipid profiles*

Table 3 shows the effect of aqueous root extracts and glibenclamide on **s**erum lipid profiles of control and experimental groups. The hyperlipidemic parameters like serum triglyceride (TG), total cholesterol (TC), and LDL cholesterol (LDLC) were increased, but HDL cholesterol decreased in diabetic groups in comparison to the normal control. However, all these parameters except HDL cholesterol were decreased significantly in the diabetic groups treated by 250 and 500 mg/Kg of AEBI or glibenclamide (0.6 mg/Kg bw) (about 39.17, 44.82 and 22.42% for total cholesterol respectively), (about 29.93, 39.31, and 32.99% for triglyceride respectively) and (about 69.63, 80.02 and 47.00% for LDL cholesterol respectively) compared to diabetic rats. HDL cholesterol was increased significantly (about 128.76, 157.95 and 143.43% respectively) compared to diabetic rats. On the other hand, normal rats group treated by 250 and 500 mg/Kg bw of AEBI and glibenclamide (0.6 mg/Kg bw) for 6 weeks, caused a little significant reduction on all parameters (about 2.45, 4.69 and 4.71% for total cholesterol respectively), (about 2.62, 8.22 and 3.25% for Triglyceride respectively and about 10.68, 18.84 and 19.25% for LDL cholesterol respectively) compared to normal control; while, the HDL cholesterol was a negligible increased (about 3.65, 11.31 and 3.26% respectively) compared to the normal control.

**Table 3:** Effect of glibenclamide and AEBI on the lipid profiles (mg/dL) in normal and diabetic rats.

**Group (n = **6)	**Treatment**	**Dose (mg/Kg)**	**Serum lipid profiles (mg/d**L)			
			**TC**	**TG**	**HDL**	**LDL**
1	N+C	10 mL/Kg	76.28 ± .98	66.42 ± 1.03	38.54 ± .48	24.25 ± 1.21
2	N+AEBI	250	74.40 ± .85	64.66 ± 1.79	39.95 ± .52	21.66 ± .93
3	N+AEBI	500	72.70 ± .54	60.96 ± .84	40.82 ± .61	19.68 ± .91
4	N+G	0.6	72.68 ± 1.02	64.26 ± .70	39.80 ± .74	19.58 ± 1.76
5	D+C	10 mL/Kg	125.94 ± 1.67^#^	121.68 ± .90^#^	14.46 ± .32^#^	87.14 ± 1.63^#^
6	D+AEBI	250	76.60 ± 1.55^d^	85.26 ± 1.87^d^	33.08 ± .76^d^	26.46 ± 1.26^d^
7	D+AEBI	500	69.48 ± 2.04a^d^	73.84 ± 1.87^d^	37.30 ± .65^d^	17.41 ± 1.73^d^
8	D+G	0.6	97.70 ± 2.49b^d^	81.53 ± .94^d^	35.20 ± .56^d^	46.18 ± 2.67^d^

AEBI: Aqueous Extract of Berberis Integerrima; N: normal; C: control; G: glibenclamide; D: diabetic; Values are presented as mean ± SEM. n = 6 in each group. One-way ANOVA followed by Tukey test. a p < 0.05 and bp < 0.01. Normal treated rats were compared with Normal control rats. #p < 0.01; Diabetic control rats were compared with Normal control rats. cp < 0.05 and dp < 0.01; Diabetic treated rats were compared with Diabetic control rats.

*Kidney parameters*

The mean values of serum urea and creatinine concentrations of both control and experimental groups are presented in Table 4. STZ-induced diabetic rats showed a significant increase (p < 0.01) in blood urea and creatinine compared to normal control. Administration of 250 and 500 mg/Kg bw of AEBI or glibenclamide (0.6 mg/Kg) led to significant decrease in serum creatinine and urea levels in STZ-induced diabetic rats (about 41.7, 52.94 and 45.29 for creatinine respectively and about 54.86, 65.56 and 62.26% for urea respectively) as compared with untreated STZ-induced diabetic rats. On the other hand normal rats treated with 250 and 500 mg/Kg bw of AEBI or glibenclamide (0.6 mg/Kg) for 6 weeks, caused a little reduction (about 3.03, 7.57 and 4.54% for creatinine respectively and about 7.04, 9.09 and 4.59% for urea respectively) compared to normal control.

**Table 4:** Effect of glibenclamide and AEBI on the liver parameters (mg/dL) in normal and diabetic rats.

**Group (n = 6)**	**Treatment**	**Dose (mg/Kg)**	**liver parameters (mg/d**L)				
			**ALP**	**ALT**	**AST**	**T bilirubin**	**T protein**
1	N+C	10 mL/Kg	93.82 ± 4.76	25.84 ± 1.60	21.00 ± 1.57	0.83 ± .04	2.06.08
2	N+AEBI	250	85.62 ± 4.15	23.28 ± 1.83	20.02 ± 099	0.74 ± .02	2.14 ± .22
3	N+AEBI	500	82.80 ± 1.59	21.10 ± 1.78	17.56 ± .99	0.76 ± .03	2.30 ± .23
4	N+G	0.6	84.06 ± 2.28	22.42 ± 2.08	19.10 ± .88	0.76 ± .03	2.17 ± .12
5	D+C	10 mL/Kg	180.78 ± 3.25^#^	48.92 ± 1.96^#^	44.68 ± 2.35^#^	1.39 ± .06^#^	0.49 ± .05^#^
6	D+AEBI	250	147.34 ± 4.56^d^	36.06 ± 1.89^d^	25.68 ± 1.79^d^	1.03 ± .07^d^	1.15 ± .05^c^
7	D+AEBI	500	101.56 ± 7.79^d^	24.28 ± 1.74^d^	22.02 ± 1.56^d^	0.82 ± .05^d^	1.80 ± .10^d^
8	D+G	0.6	112.94 ± 5.48^d^	28.06 ± 1.48^d^	22.20 ± .65^d^	0.97 ± .09^d^	1.12 ± .08^c^

AEBI: Aqueous Extract of Berberis Integerrima; N: normal; C: control; G: glibenclamide; D: diabetic; Values are presented as mean ± SEM; n = 6 in each group. One way ANOVA followed by Tukey test. a p < 0.05 and bp < 0.01; Normal treated rats were compared with Normal control rats. #p < 0.01; Diabetic control rats were compared with Normal control rats. cp < 0.05 and dp < 0.01; Diabetic treated rats were compared with diabetic control rats

*Liver parameters*

Table 5 shows the mean values of AST, ALT, ALP activities and serum total bilirubin and total protein levels of both control and experimental groups after 6 weeks. In STZ-induced diabetic rats, the activities of blood AST , ALT, ALP and the serum total bilirubin level were significantly increased (p < 0.01), but serum total protein levels were decreased (p < 0.01) compared to their normal levels. On the other hand, treatment of the STZ-induced diabetic rats by 250 and 500 mg/Kg of AEBI or glibenclamide (0.6 mg/Kg) caused a significant reduction in the activity of these parameters in blood by 42.52, 50.71 and 50.31%, respectively for AST and by 26.28, 50.36 and 42.64% respectively for ALT, and by 18.49, 43.82 and 37.52% respectively for ALP and by 25.89, 41 and 30.21% respectively for serum total bilirubin level but increased in blood total protein by 128.57, 267.34 and 134.69% compared to the mean values of untreated diabetic group. On the other hand, normal rats group treated by 250 and 500 mg/Kg bw of AEBI or glibenclamide (0.6 mg/Kg bw) for 6 weeks, caused a little reduction (about 4.66, 16.33 and 9.04 for AST respectively), (about 9.90, 18.34 and 13.23 for ALT respectively), (about 8.74, 11.74 and 10.40 for ALP respectively), (about 10.84, 8.43 and 8.43 for bilirubin respectively) and also a little increase in total protein (about 3.88, 11.65 and 5.33 respectively) compared to normal control.

**Table 5:** Effects of glibenclamide and AEBI on kidney parameters (mg/dL) in normal and diabetic rats

**Group (n = 6)**	**Treatment**	**Dose (mg/Kg)**	**Kidney parameters (mg/dL)**	
			**Serum creatinine**	**Serum urea**
1	N+C	10 mL/Kg	0.66 ± .02	19.58 ± 1.24
2	N+AEBI	250	0.64 ± .02	18.20 ± 1.08
3	N+AEBI	500	0.61 ± .00	17.80 ± 1.37
4	N+G	0.6	0.63 ± .01	18.68±1.54
5	D+C	10 mL/Kg	1.70 ± .10^#^	69.70±1.83^#^
6	D+AEBI	250	1.00 ± .13^d^	31.46±2.45^d^
7	D+AEBI	500	0.80 ± .14^d^	24.00±1.43^d^
8	D+G	0.6	0.93 ± .06^d^	26.30±1.36^d^

AEBI: Aqueous Extract of Berberis Integerrima; N: normal; C: control; G: glibenclamide; D: diabetic; Values are presented as mean ± SEM. n = 6 in each group. One way ANOVA followed by Tukey test. a: p < 0.05 and b: p < 0.01 Normal treated rats were compared with Normal control rats. #p < 0.01; Diabetic control rats were compared with Normal control rats. c: p < 0.05 and d: p < 0.01; Diabetic treated rats were compared with Diabetic control rats

## Discussion

In the present study, the blood glucose level had significant increase in diabetic rats. This result is similar to the finding of Augusti *et al. *and Campos *et al. *in rats (15, 16), Kumar and Reddy in mice (17) and Jain and Vyas in rabbits (18). There are many studies in this regard that indicating the effective reduction in blood sugar levels in STZ-induced diabetic rats (19-21). In the present study, the AEBI (dose dependently) and glibenclamide (0.6 mg/Kg) showed a significant hypoglycemic activity in normoglycemic in 2 weeks of treatment, however, at the end of the 6^th^ week, it was increased to achieve the normal blood sugar levels. Injection of STZ (65 mg/Kg, IP) led to about 3.5-fold elevation of fasting blood glucose levels. Diabetic rates treated by AEBI (dose dependently) or glibenclamide showed a significantly decrease in blood glucose level during 6 weeks of treatment in comparison with diabetic control. The activity of this fraction could be due to the presence of Berberine, saponins, flavonoids, alkaloids and steroids components. Different mechanisms already exist to use the plant extracts for reducing the blood glucose. Based on the results, it is assumed that the root extract could be responsible for the stimulation insulin release and the observed restoration of metabolic activities. In addition, these results indicated that the extracts possess active phytochemical principles with either cytoprotective functions on the pancreatic *β*-cells or insulino-protective properties. The hypoglycemic activity in a number of other plants is applied through the stimulation of insulin release (22, 23). The hypoglycemic activity in some of other plants is similar to the well-known sulfonylurea drugs like glibenclamide. They reduce blood glucose in normoglycemic animals (24, 25). The glibenclamide effects on blood glucose levels by increasing the activity of pancreatic *β*-cells of the pancreas result in the secretion of a large amount of insulin (26, 27). The maximum reduction in serum glucose levels was seen in AEBI at dose of 500 mg/Kg (Table 1) hence, we could say that aqueous root extract of Berberis integerrima had a beneficial effect on carbohydrate.

In addition, results of the present study showed that diabetic rats exhibited a significant decrease in body weight after 6 weeks. These results are in agreement with those previously obtained (28-30). Body weight loss in diabetic rats may be due to the fats and protein catabolism, even though the food intake is more in diabetic rats than control group. Due to the insulin deficiency protein, content is decreased in muscular tissue by proteolysis (31). Fat mobilization in skeletal muscle in STZ-induced diabetic rats (32) significantly induces the weight loss (33) as observed in the present study. However, diabetic rates treated by 250 and 500 mg/Kg of AEBI or glibenclamide (0.6 mg/Kg bw) showed a little reduction in body weight which may be explained by the increased insulin secretion or increased food consumption (34, 35).

Lipids play a major role in the disorder of diabetes mellitus. The most common lipid disorders in diabetes are hypertriglyceridemia and hypercholesterolemia (36). In our study, we have noticed significantly increased levels of serum total cholesterol, triglycerides and LDL-cholesterol but markedly decreased level of serum HDL-cholesterol in STZ-induced diabetic rats. These results are in agreement with those obtained (37-39). The main reason for the abnormal concentration of serum lipids in diabetic rats is an increase in the mobilization of free fatty acids from peripheral fat depots, since insulin inhibits the hormone-sensitive lipase (40). Excessed fatty acids in the serum of diabetic rats are converted into phospholipids and cholesterol in the liver. These two substances along with excessed triglycerides formed at the same time in the liver maybe discharged into the blood in the form of lipoproteins (41). The present study showed that AEBI (250 and 500 mg/Kg) or glibenclamide (0.6 mg/Kg bw) had favorably modified serum lipid profile in rats with significant decreases (dose-dependently for AEBI) in total cholesterol, LDL-cholesterol, triglycerides and increased the serum HDL-C level in diabetic rats. These findings are consistent with earlier report that showed the administration of *Berberis vulgaris *Fruit Extract (3 g/Kg/d) for 3 months in type 2 diabetic patients decreased the Serum glucose, Triglycerides, total cholesterol and LDL-c and also increased the HDL-c (53).

The mechanism(s) of the hypolipidemic actions of AEBI are not known; however, they could be mediated by controlling tissue metabolism and improved insulin secretion and action since insulin lowers the lipid levels and normalizes lipids in STZ-induced diabetic rats (42).

In our study, we have noticed significantly increased levels of serum urea and creatinine in STZ-induced diabetic rats. Many studies have shown a significant increase in the rate of kidney cell damage (nephropathy) in diabetes disorders (43). Finally, this nephropathy reduces the physiological function and changes in the structure of kidney in diabetes (44). Hyperglycemia increases the generation of free radicals by glucose auto-oxidation and the increment of free radicals may lead to kidney cells damage (45). On the other hand, studies have shown that the increase in oxidative stress the basement membrane of glomerular is thickening and the mesenchymal expansied (46). These changes lead to hypertrophy and decrease in physiological function of the kidney as well as increase in serum urea and creatinine levels (47). Our findings also show these changes. Previous changes in serum urea and creatinine concentrations strongly suggested impairment of kidney function in diabetes. The treatment with AEBI especially at dose of 500 mg/Kg and glibenclamide (0.6) return these parameters close to normal levels. The main effect of the AEBI and glibenclamide is presumably due to its ability to increase the insulin secretion since insulin lowers glucose levels and normalizes glucose in STZ-induced diabetic rats (22, 23).

The enzymes levels such as AST and ALT are largely used to assess the liver damage by streptozotocin. Necrosis or membrane damage releases the enzyme into circulation and hence it can be measured in the serum. High levels of AST indicates liver damage that occurs in many diseases such as viral hepatitis as well as cardiac infarction and muscle injury. AST catalyses the conversion of alanine to pyruvate and glutamate and is released in a similar manner. Therefore, ALT is more specific to the liver, and thus is a better parameter for detecting the liver injury. Elevated levels of serum enzymes indicate cellular leakage and functional integrity loss of cell membrane in liver (48). Serum ALP, bilirubin and total protein levels, on the other hand, are related to the function of hepatic cell. Increase in serum level of ALP is due to increased synthesis, in presence of increasing biliary pressure (49). Decline in serum total protein may be due to the inhibited oxidative phosphorylation process which leads to a decrease in protein synthesis, an increase in the catabolic process and reduction of protein absorption (50). STZ-induced caused a significant (p < 0.001) elevation in enzyme levels such as AST, ALT, ALP, level total bilirubin and decrease in total protein when compared to control. There was a significant (p < 0.001) restoration of these enzyme levels on the administration of AEBI in a dose-dependent manner and also by glibenclamide at dose of 0.6 mg/Kg. The reversal of increased serum enzymes in STZ-induced liver damage by the extract may be due to the prevention of the leakage of intracellular enzymes by its membrane stabilizing activity. This is in agreement with the commonly accepted view that serum levels of transaminases return to normal with the healing of hepatic parenchyma and the regeneration of hepatocytes (51). Effective control of ALP, bilirubin and total protein levels point toward an early improvement in the secretary mechanism of the hepatic cells. The efficacy of any hepatoprotective drug is dependent on its capacity of either reducing the harmful effect or restoring the normal hepatic physiology that has been distributed by a hepatotoxin. Both glibenclamide and the aqueous extract of *Berberis integerrima *root decreased the STZ-induced elevated enzyme levels in tested groups, indicating the protection of structural integrity of hepatocytic cell membrane or regeneration of damaged liver cells.

## Conclusion

The aqueous extract of *Berberis integerrima *root exhibited significant antihyperglycemic, antihyperlipidemic and antioxidant activities in STZ-induced diabetes. These effects of root extract of *Berberis integerrima *especially at dose of 500 mg/Kg in all parameters except for the blood glucose, (similar) are more than glibenclamide in diabetic rats. The results support the use of this extract in traditional medicine, although the total extract does not confer any additional benefits or disadvantages compared with the well-known sulfonylurea drugs like glibenclamide.
